# Evidence and assessment of parenchymal patterns of ultrasonography for breast cancer detection among Chinese women: a cross-sectional study

**DOI:** 10.1186/s12880-021-00687-0

**Published:** 2021-10-19

**Authors:** Zhongtao Bao, Yanchun Zhao, Shuqiang Chen, Xiaoyu Chen, Xiang Xu, Linglin Wei, Ling Chen

**Affiliations:** 1 Department of Ultrasound, First Affiliated Hospital of Fujian Medical University, No 20 Cha zhong Road, Taijiang District, Fuzhou, 350000 Fujian China; 2 Department of Ultrasound, Provincial Clinical Academy of Fujian Medical University, Fujian Provincial Hospital, Fuzhou, 350001 Fujian China

**Keywords:** Breast cancer, Breast cancer risk, Breast density, Breast cancer screening, Breast parenchymatous tissue, Chinese women, Ultrasound

## Abstract

**Background:**

Screening of breast cancer in asymptomatic women is important to evaluate for early diagnosis. In China ultrasound is a more frequently used method than mammography for the detection of breast cancer. The objectives of the study were to provide evidence and assessment of parenchymal patterns of ultrasonography for breast cancer detection among Chinese women.

**Methods:**

Breast ultrasound examinations including the parenchymatous pattern of cytopathological confirmed breast cancer (n = 541) and age-matched cytopathological not confirmed breast cancer (n = 849) women were retrospectively reviewed by seven sonographer physicians. According to compositions of ducts, the thickness of the breast, diameter of ducts, fat lobules, and fibro glandular tissues, the breast parenchymatous pattern was categorized into heterogeneous (high percentage of fatty tissues), ductal (the inner diameters of ducts > 50% of the thick mass of the breast), mixed (the inner diameters of ducts was 50% of the thick mass of the breast), and fibrous categories (a dense classification of the breast).

**Results:**

Heterogeneous (*p* < 0.0001, OR = 3.972) and fibrous categories (*p* < 0.0001, OR = 2.702) were higher among women who have cytopathological confirmed breast cancer than those who have not cytopathological confirmed breast cancer. The heterogeneous category was high-risk ultrasonographic examination category followed by the fibrous category. Agreements between sonographer physicians for categories of ultrasonic examinations were fair to good (Cohen’s *k* = 0.591).

**Conclusions:**

Breast cancer risk in Chinese asymptomatic women differ according to the ultrasonographic breast parenchymal pattern.

*Level of Evidence*: III.

*Technical efficacy stage*: 2.

## Background

Breast cancer is the most common in Chinese women [1, 2]. Screening of breast cancer in asymptomatic women is important to evaluate for the early diagnosis [3]. Considering various risks for breast cancer, various breast cancer assessment models are developed to identify the risk of breast cancer [1] but there is a lack of a consistent breast cancer assessment model for Chinese women [4]. A proper screening method of breast cancer for Chinese women is necessary [5].

Genetic and environmental factors affect breast parenchymatous tissue structure and there is a correlation between breast parenchymatous tissue structure and the risk of breast cancer in any individual woman [6]. Therefore, it is possible to predict the breast cancer risk of a healthy woman by imaging modalities like mammography and ultrasound.

Mammography is an established imaging method for breast cancer screening because it has accuracy but has low sensitivity [7]. This is because the mammary parenchyma of women is different due to different parameters [8]. Also, mammography is successful among Caucasian women [3]. However, Chinese women have comparatively smaller and dense breasts and have greater parenchymal density than Caucasian women [9]. Mammography has lower sensitivity for dense tissues of the breast [10] and ultrasound is a cost-effective and more sensitive imaging method for dense tissues of the breast [11]. Therefore, in China ultrasound is a more frequently used method than mammography for the detection of breast cancer [3]. The Chinese government is started breast cancer screening by ultrasound in rural areas [3].

The objectives of retrospective analysis of cross-sectional study were to provide evidence and assessment of breast parenchymal patterns of ultrasonography for breast cancer detection among Chinese women considering those of women with cytopathology confirmed breast cancer as a reference standard.

## Methods

### Inclusion criteria

Women who underwent ultrasound examinations for breast cancer screening were included in the analyses.

### Exclusion criteria

Women who underwent breast cancer surgeries, estrogen replacement therapy, and have incomplete data in the institutional records were excluded from the study.

### Ultrasound examination

In a lateral or supine position with raised arms, fully exposed breasts, and axillae conditions of women, ultrasound examinations were performed. EPIQ 5 (Koninklijke Philips N.V., Amsterdam, Netherlands), M8 (Mindray Medical International Limited, Nanshan, Shenzhen, China), and iU22 (Philips, Amsterdam, Netherlands) ultrasound devices were preferred to examine women. Linear array probes for 5–13 MHz, 6.6–13 MHz, and 7.5–10 MHz respectively were used. All examinations were performed by seven sonographer physicians (minimum 3-years experiences of institutes). Multi-angle and multi-plane ultrasound scans were performed for bilateral breasts and axillae. Two mutually orthogonal planes were used for confirmation of all lesions. The characteristics of the breast parenchyma and ultrasound images of all lesions were recorded. The images were taken at the same location in each breast.

### Breast biopsies

Ultrasound-guided core biopsies were performed using an 18 G needle (BD Biosciences, Chicago, IL, USA) [12]. Core biopsies were performed by pathologists (minimum 3-years of experience of institutes).

### Breast tissues histopathology

Breast tissue histopathology was performed by pathologists (minimum 3-years of experience of institutes). The diagnosis of breast cancer was confirmed by breast tissue histopathology.

### Ultrasonographic classification

**Fig. 1:**
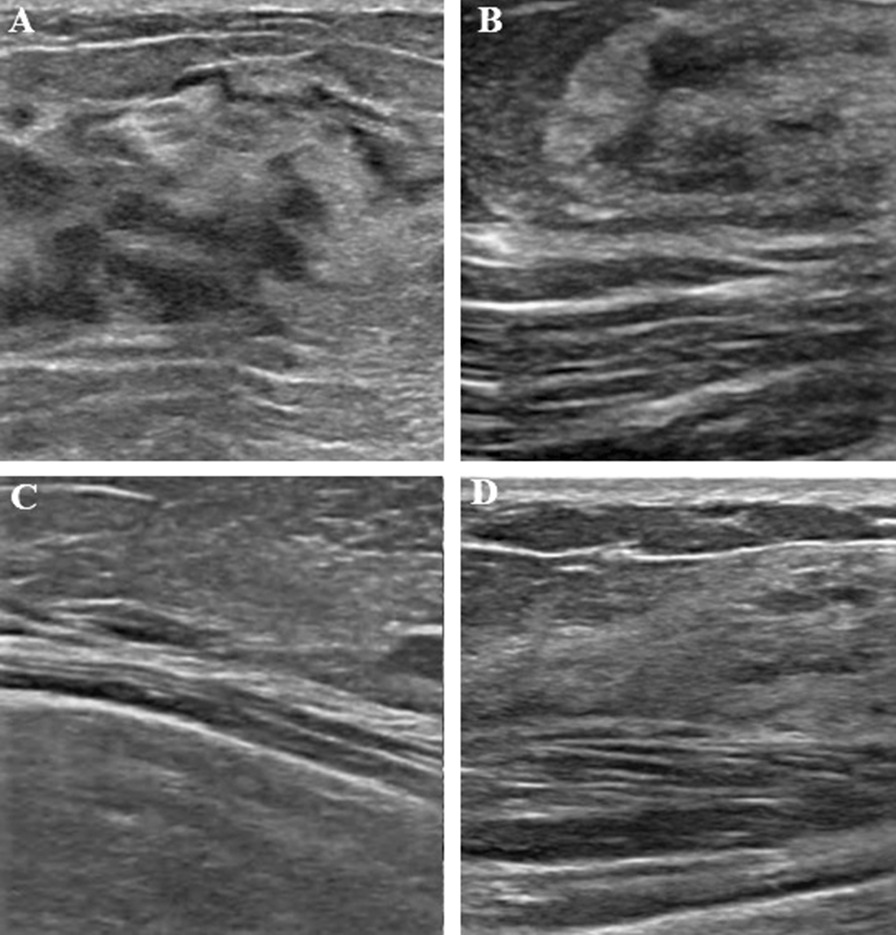
Ultrasonographic classification. **a** heterogeneous category (high percentage of fatty tissues), **b** ductal category (the inner diameters of ducts > 50% of the thick mass of the breast), **c** Mixed category (the inner diameters of ducts was 50% of the thick mass of the breast), and **d** Fibrous category (a dense classification of the breast)

The thickness of the breast, ducts, fat lobules, and fibro glandular tissues were examined by ultrasonography. According to compositions of ducts, the thickness of the breast, diameter of ducts, fat lobules, and fibro glandular tissues, the breast parenchymatous pattern was categorized into 4 types: heterogeneous category: the inner diameters of ducts were less than 50% of a thick mass of the breast, the hypo-echoic fatty mass of the breast, less dense iso-echoic fibro glandular tissue, and hypo-echoic ductal ingredients. Ductal category: the inner diameters of ducts was more than 50% of the thick mass of the breast, hypo-echoic ductal ingredients in the mesh structure. Mixed category: the inner diameters of ducts was 50% of the thick mass of the breast, iso-echoic fibro glandular tissue, and hypo-echoic ductal ingredients. Fibrous category: the inner diameters of ducts was less than 50% of the thick mass of the breast, dense iso-echoic fibro glandular tissue, and less hypo-echoic ductal ingredients [3]. The different breast parenchymatous pattern categories are presented in Fig. 1.

### Statistical analysis

SPSS 26.0 IBM Corporation, Armonk, NY, USA was used for statistical analyses. One-way analysis of variance (ANOVA) was used for categorical data and the Fischer exact test or Chi-square test of Independence was used for continuous data. All results were considered significant if *p* < 0.05, odds ratio (OR) with 95% of confidence interval (Cl). Cohen’s *k* was evaluated for agreements among sonographer physicians. Values were 0 ≤ *k* ≥ 0.4: poor agreement, 0.41 ≤ *k* ≥ 0.75: fair to good agreement, 0.76 ≤ *k* > 1.0: excellent agreement, *k* = 1.0: perfect agreement [13].

## Results

### Study population

**Fig. 2:**
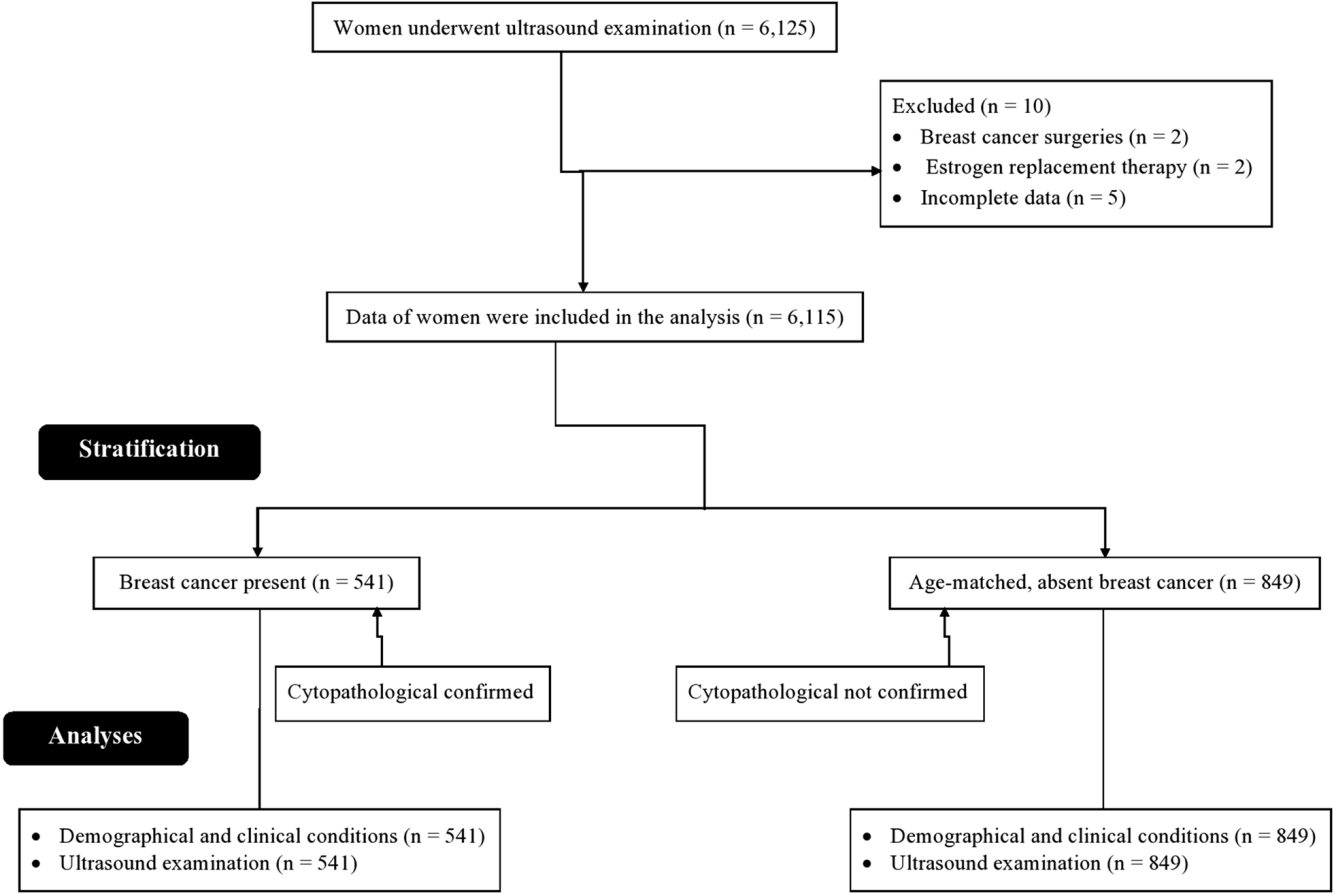
Flow diagram of the study

A total of 6,125 Chinese women have undergone ultrasound examinations at the First Affiliated Hospital of Fujian Medical University, Fuzhou, Fujian, China and the Fujian Provincial Hospital, Fuzhou, Fujian, China from 15 April 2015 to 13 May 2019. Three women underwent breast cancer surgeries, two women underwent estrogen replacement therapy, and five women have no complete data in the institutional records. Therefore, data of these women (n = 10) were excluded from the study. Among 6,115 women, 541 women had cytopathological confirmed breast cancer. From 5,574 cytopathological not confirmed breast cancer women, data of 849 age-matched women were randomly selected for the study. The demographical, social, and clinical conditions of women are presented in Table 1. The flow diagram of the study is presented in Fig. 2.

**Table 1:** The demographical, social, and clinical conditions of women who underwent ultrasound examination and selected for study

Parameters		Cytopathological did not confirm breast cancer women	Cytopathological confirmed breast cancer women	Comparisons
Women		849	541	*p*-value
Age at examination (years)	Minimumact	27	25	0.221
	Maximum	72	71	
	Mean ± SD	50.12 ± 11.19	49.48 ± 15.14	
Ethnicity	Han Chinese	787 (92.88)	506 (93.8)	0.915
	Mongolian	52 (6)	29 (5)	
	Tibetan	9 (1)	5 (1)	
	Uighur Muslim	1 (0.12)	1 (0.2)	
Menopausal status	Premenopausal	520 (61)	335 (62)	0.821
	Postmenopausal	329 (39)	206 (38)	
Body mass index (kg/ m^2^)	< 25	625 (74)	403 (74)	0.751
	≥ 25	224 (26)	138 (26)	
Family history of breast cancer	Yes	48 (6)	32 (6)	0.906
	No	801 (94)	509 (94)	

Categorial data are demonstrated as frequency (percentages) and continuous data are mean ± SD (standard deviation)

One-way ANOVA for categorical data and Fischer exact test or Chi-square test of Independence for continuous data were performed for statistical analyses

All results were considered significant if the *p*-value less than 0.05

### Ultrasound examination

Heterogeneous and fibrous categories were higher among women who have cytopathological confirmed breast cancer than those who have not cytopathological confirmed breast cancer (*p* < 0.0001 for both). However, mixed type and ductal categories were the same among women who have cytopathological confirmed breast cancer and those who have not cytopathological confirmed breast cancer. The breast parenchymatous pattern categories shown in ultrasound examinations are reported in Table 2. Heterogeneous and fibrous categories were high-risk ultrasonographic examination categories for breast cancer. The OR is highest for the heterogeneous category followed by the fibrous category.

**Table 2:** Ultrasound examination

Parenchymatous Category	Cytopathological confirmed breast cancer women	Cytopathological not confirmed breast cancer women	Comparisons		
Women	541	849	OR	95% Cl	*p*-value
Heterogeneous	49 (9)*	21 (2)	3.927	2.327–6.627	< 0.0001
Ductal	29 (5)	65 (8)	0.683	0.435–1.073	0.101
Mixed	168 (31)	502 (59)	0.311	0.248–0.391	< 0.0001
Fibrous	295 (55)*	261 (31)	2.702	2.161–3.378	< 0.0001

Variable are demonstrated as frequency (percentages)

Fischer exact test was performed for statistical analyses

All results were considered significant if the *p*-value was less than 0.05 and OR more than 1

OR: Odd ratio,

Cl: Confidence interval

^*^Significantly higher value

### Agreements among sonographer physicians

Agreements among sonographer physicians for categories of ultrasonic examinations were fair to good (Table 3).

**Table 3:** Agreements among sonographer physicians

Parameters	Values
Sonographer physicians	07
*k*	0.591
95% Cl	0.541–0.721

Cl: Confidence interval

0 ≤ *k* ≥ 0.4: poor agreement, 0.41 ≤ *k* ≥ 0.75: fair to good agreement, 0.76 ≤ *k* > 1.0: excellent agreement, *k* = 1.0: perfect agreement

## Discussion

The current study reported that heterogeneous and fibrous categories were high-risk ultrasonographic examination categories for breast cancer. The results of the breast parenchymatous pattern categories of the current study were agreed with those of an available study [3], retrospective studies [14, 15], and case–control study [16]. The heterogeneous category in the current study was included a high percentage of fatty tissues. The higher amount of breast fatty tissue independent of the other risk factors is associated with the risk of breast cancer [16]. The fibrous category in the current study was included a dense classification of the breast. Atypical epithelial hyperplasia is associated with the risk of breast cancer [17]. The distribution of ultrasonographic parenchymatous patterns is different between women who have cytopathological confirmed breast cancer and those who have not cytopathological confirmed breast cancer.

The study reported fair to good agreements among sonographer physicians for categories of ultrasonic examinations. The results of agreements between sonographer physicians for categories of ultrasonic examinations were parallel with those of an available study [3] and prospective study [13]. The use of three different ultrasound machines, seven sonographer physicians as reader, and the use of manual reading of ultrasound images were responsible for the lower agreement.

There are several limitations of the study, for example, the correlation between ultrasonographic parenchymatous pattern and mammographic parenchymatous pattern were not discussed. The possible justification is that by adding mammography there was an addition of extra cost on women’s heads. The retrospective nature of the study and lack of prospective study.

## Conclusions

Breast ultrasound examinations are associated with breast cancer in asymptomatic women. Heterogeneous and fibrous categories of parenchymatous pattern of breast tissues were high-risk ultrasonographic examination categories for breast cancer. The ultrasonographic parenchymatous pattern could be used for screening breast cancer in asymptomatic Chinese women. If questionnaires and ultrasound examinations were performed in the early stage of breast cancer, it is possible to control the spoil of life of women due to breast cancer. The study data have the potential for the basis of breast cancer risk prediction.

## Data Availability

The datasets were used and analyzed during the current study available from the corresponding author on reasonable request.

## Abbreviations

ANOVA: Analysis of variance
OR: Odds ratio
Cl: Confidence interval
SD: Standard deviation
